# The Impact of Microbiome Dysbiosis on Hematopoietic Stem Cell Transplantation Outcomes: A Review Article

**DOI:** 10.7759/cureus.63995

**Published:** 2024-07-06

**Authors:** Zeyad Khalil, Soheir Maher

**Affiliations:** 1 General Medicine, October 6 University, Cairo, EGY; 2 Emergency Medicine, Al-Fares Crystal Medical Complex, Makka, SAU

**Keywords:** short-chain fatty acids, antibiotic stewardship, graft-versus-host disease, probiotics, hematopoietic stem cell transplantation, microbiome diversity

## Abstract

Microbiome dysbiosis has emerged as a critical factor influencing the outcomes of hematopoietic stem cell transplantation (HSCT). This comprehensive review delves into the intricate relationship between microbiome composition and HSCT outcomes, highlighting the mechanisms through which dysbiosis impacts engraftment, graft-versus-host disease (GVHD), infection rates, and overall survival. The gut microbiome plays a pivotal role in modulating immune responses and maintaining intestinal homeostasis, both of which are crucial for the success of HSCT. This review aims to elucidate the underlying pathways and potential therapeutic strategies to mitigate adverse outcomes associated with microbiome imbalances in HSCT patients. Integrating microbiome modulation strategies such as probiotics, prebiotics, fecal microbiota transplantation (FMT), and antibiotic stewardship into clinical practice can significantly improve patient outcomes and quality of life post-transplantation.

## Introduction and background

Hematopoietic stem cell transplantation (HSCT) is a cornerstone treatment for various hematologic malignancies, severe aplastic anemia, and certain genetic disorders [1,2]. The procedure involves the infusion of hematopoietic stem cells from a compatible donor to restore the recipient’s bone marrow function after myeloablative therapy [1,2]. Despite its curative potential, HSCT is fraught with complications that can significantly impact patient morbidity and mortality. Among these complications, graft-versus-host disease (GVHD), infections, and poor engraftment are the most formidable challenges [1,2].

Recent advancements in microbiome research have shed light on the profound impact of the gut microbiome on HSCT outcomes. The human microbiome, consisting of trillions of microorganisms residing in the gut, skin, and other body sites, plays a crucial role in maintaining immune homeostasis and overall health [3]. Dysbiosis, or the disruption of this microbial balance, has been implicated in adverse HSCT outcomes. Studies have demonstrated that patients with higher gut microbial diversity exhibit better engraftment rates and reduced time to neutrophil recovery [4,5]. Conversely, reduced microbial diversity has been associated with increased incidences of GVHD and infections, significantly impacting overall survival [6].

The gut microbiome, in particular, is integral to the regulation of immune responses and the maintenance of intestinal barrier function, both of which are critical for successful HSCT [7]. Specific gut bacteria, such as *Blautia *and *Lactobacillus*, have protective effects against GVHD by promoting regulatory T cell (Treg) expansion and reducing pro-inflammatory cytokines [8,9]. Conversely, the loss of microbial diversity and the overgrowth of pathogenic bacteria like *Enterococcus* are linked to increased GVHD severity and higher infection rates [10-12].

## Review

A healthy microbiome and intestinal homeostasis

The human microbiome, a diverse collection of bacteria, viruses, archaea, and eukaryotic microbes, primarily resides in the gut [9]. Bacteria form the largest group within the intestinal microbiome, with most species belonging to the Firmicutes or Bacteroidetes phyla, alongside smaller contributions from the Proteobacteria, Actinobacteria, Fusobacteria, and Verrucomicrobia phyla [11]. This dynamic ecosystem within the intestinal tract is continuously influenced by environmental changes such as diet, medication, and disease, which can dramatically alter microbial composition [11]. Factors crucial for maintaining homeostasis and preventing pathogen outgrowth and translocation include an intact gut epithelium that physically separates luminal microorganisms from underlying host tissue and a mucus layer produced by goblet cells that prevent bacterial translocation and mucosal barrier injury [9,11,12]. Anaerobic commensals in the gut microbiota community provide resistance against pathogen colonization, and their products further prevent pathogen outgrowth [13-29,21]. Molecules derived from commensal bacteria signal to Paneth cells via toll-like receptors, inducing the production of antimicrobial peptides such as defensins and regenerating gene proteins, which shape microbial composition by sequestering essential nutrients and permeabilizing bacterial membranes [13]. These commensals also produce essential metabolites for the human host, such as vitamins, short-chain fatty acids (SCFA), aryl hydrocarbon receptor ligands, and secondary bile acids [17-19,24]. These metabolites are crucial for maintaining epithelial integrity, stimulating the mucosal immune system, and exerting effects on peripheral organs via systemic circulation [17-19]. Short-chain fatty acids serve as an energy source for colonocytes, promote mucus secretion, enhance tight junction protein expression, induce anti-inflammatory cytokines, and promote differentiation of regulatory T cells via engagement with G-protein coupled receptors on epithelial and immune cells [21-24]. Indoles, another set of metabolites, induce IL-22 production by aryl hydrocarbon receptor-expressing lymphocytes, enhancing epithelial barrier function and promoting antimicrobial peptide production [25,26].

Microbiome and transplant-related toxicities

During the peri-transplant period, multiple factors disrupt the intestinal microbiota, including conditioning, diet changes, and antibiotic exposure. Early post-transplant, there is a loss of microbial diversity and a shift towards microbiomes dominated by *Enterococci*, *Streptococci*, or Proteobacteria, reducing the presence of anaerobic populations like *Bacteroides*,* Clostridium*, and *Bifidobacterium* [25,28]. Intestinal domination, defined as a relative abundance of ≥30% of a single taxonomic unit, is linked to antibiotic exposure and increases the risk of bloodstream infection by the dominating taxon [28]. Reductions in fecal α-diversity can be observed before hematopoietic cell transplantation (HCT) in some patients and worsen post-transplant, correlating with antibiotic treatment timing and type [29]. Clinical studies show a reduction in bacterial burden and fecal α-diversity following cancer treatment, consistent with pre-transplant dysbiosis [29-31]. A large observational study involving 8,767 stool samples from 1,362 patients across different geographic locations documented a significant association between intestinal diversity during the peri-engraftment period and transplant outcomes [31,32]. Low microbial diversity was associated with higher transplantation-related mortality, GVHD-related mortality, and lower overall survival. Intestinal domination, particularly by *Enterococcus*, was prevalent in over 75% of samples collected a week post-transplant and was associated with lower survival and higher GVHD-related mortality [32,33]. Acute GVHD (aGVHD) patients show pronounced microbiome perturbations, with pre-clinical studies in mice demonstrating the critical role of intestinal microbiota in aGVHD development and vice versa [21,33]. Dysbiotic microbiomes in IL-17-deficient mice induced hyperacute GVHD, and antibiotic-mediated bacterial depletion prevented lethal aGVHD by inhibiting MHCII expression upregulation on intestinal epithelial cells [33]. Graft-versus-host disease-mediated tissue damage, including Paneth cell destruction, further propagates pathogenic bacterial expansion, accelerating GVHD [33]. The abundance of *Enterococcus* species is associated with severe aGVHD, whereas the presence of butyrate-producing *Blautia *offers protective capacities against aGVHD in mice and humans [33]. Changes in gut microbiota composition are linked not only to aGVHD but also to other transplant-related complications, such as decreased relapse risk or progressive disease with certain bacterial clusters, and lower rates of viral lower respiratory tract infections with butyrate-producing bacteria presence [33].

Microbiome and engraftment relationship

The gut microbiome plays a pivotal role in the engraftment process during HSCT, with its diversity and composition critically influencing transplant outcomes [3]. Studies have shown that higher microbial diversity in the gut is associated with better engraftment rates and faster neutrophil recovery [4]. The mechanisms underlying these effects are complex and involve multiple pathways, including the production of SCFAs by beneficial bacteria like Bacteroides and Firmicutes. Short-chain fatty acids, including acetate, propionate, and butyrate, are metabolites produced through the fermentation of dietary fibers by gut microbiota [4,5]. These metabolites play several crucial roles: they strengthen the intestinal barrier by promoting the production of tight junction proteins, which prevent translocation of pathogens and endotoxins that can cause systemic inflammation; they have anti-inflammatory properties, binding to G-protein coupled receptors (GPCRs) such as GPR41 and GPR43 on immune cells, leading to the activation of anti-inflammatory pathways, and enhancing the differentiation of regulatory T cells (Tregs), which are vital for maintaining immune tolerance and preventing graft-versus-host disease (GVHD); and they influence hematopoietic stem cell (HSC) function by providing energy sources and promoting the proliferation and differentiation of these cells [6].

Certain bacterial taxa are crucial for positive transplant outcomes. For instance, Bacteroides and Firmicutes not only produce beneficial SCFAs but also contribute to the overall health of the gut microbiome [6]. The presence of these bacteria has been linked to better immune function and reduced inflammation. Besides SCFAs, other microbial metabolites also play significant roles in the engraftment process. Indole derivatives, produced from tryptophan metabolism, activate the aryl hydrocarbon receptor (AhR) on intestinal epithelial cells and immune cells, promoting mucosal barrier function and immune regulation, which are crucial for preventing GVHD and supporting engraftment [7]. Also, secondary bile acids, metabolized by gut bacteria from primary bile acids, modulate immune responses and influence the differentiation and proliferation of HSCs [7]. The use of broad-spectrum antibiotics during HSCT can significantly disrupt the gut microbiome, reducing its diversity. This disruption is associated with poorer engraftment rates and increased severity of GVHD [7]. Antibiotics can diminish beneficial bacterial populations, leading to an imbalance that favors pathogenic bacteria and inflammatory responses. Overall, the gut microbiome's influence on HSCT engraftment is multifaceted, involving direct effects on HSCs, modulation of immune responses, and maintenance of gut barrier integrity [8].

Microbiome and infection

Infections are a leading cause of morbidity and mortality in HSCT recipients [9]. The gut microbiome acts as a crucial barrier against pathogenic microorganisms. Dysbiosis, an imbalance in the gut microbiota, compromises this barrier, facilitating the translocation of pathogens and increasing the risk of bloodstream infections [9]. Several mechanisms are involved in this process. The gut microbiome maintains the integrity of the intestinal mucosa by promoting the production of mucus and tight junction proteins, which prevent pathogen translocation [10]. Dysbiosis weakens this barrier, allowing bacteria and endotoxins to enter the bloodstream and cause infections [10]. The gut microbiome plays a key role in modulating the host immune system. Beneficial bacteria stimulate the production of antimicrobial peptides and the differentiation of immune cells such as regulatory T cells (Tregs) [11]. Dysbiosis leads to impaired immune responses and increased susceptibility to infections [10-12]. A healthy microbiome competes with pathogenic bacteria for nutrients and space, preventing their overgrowth [11]. Dysbiosis disrupts this balance, allowing pathogenic bacteria to proliferate and cause infections [12]. Dysbiosis can be caused by host-specific factors such as genetic background, health status (infections, inflammation), and lifestyle habits or, more importantly, by environmental factors such as diet (high sugar, low fiber), xenobiotics (antibiotics, drugs, food additives), and hygiene [13]. The use of broad-spectrum antibiotics in HSCT patients exacerbates dysbiosis, further increasing infection susceptibility [13-16]. Antibiotics can significantly reduce microbial diversity and deplete beneficial bacterial populations, creating an environment conducive to the growth of opportunistic pathogens such as *Clostridioides difficile*, *Enterococcus*, and multidrug-resistant Gram-negative bacteria [16]. Moreover, specific pathogens such as *Enterococcus* have been found to correlate with adverse outcomes in HSCT recipients [16]. Studies have shown that gut domination by *Enterococcus* is associated with an increased risk of bloodstream infections and severe GVHD [17]. This highlights the importance of maintaining a balanced gut microbiome to prevent infections and improve transplant outcomes [17]. In summary, the gut microbiome plays a vital role in protecting HSCT recipients from infections [18]. Dysbiosis compromises this protective barrier, leading to increased infection risk. Strategies to preserve or restore a healthy microbiome, such as judicious use of antibiotics, dietary interventions, probiotics, prebiotics and synbiotics, and potentially fecal microbiota transplantation, may help reduce infection-related morbidity and mortality in HSCT patients [18].

Probiotics and prebiotics

The administration of probiotics and prebiotics aims to restore microbiome balance and improve HSCT outcomes [13]. Probiotics such as *Bifidobacterium* have shown promise in reducing GVHD incidence and severity [13]. For example, *Bifidobacterium longum* can modulate the immune system and enhance gut barrier function, reducing inflammation [13]. Prebiotics, which are non-digestible food ingredients like inulin that promote the growth of beneficial bacteria, can also help restore microbial diversity [13]. Fecal microbiota transplantation (FMT) involves the transfer of fecal bacteria from a healthy donor to a recipient, aiming to restore a balanced and diverse gut microbiota [14]. This is particularly crucial in HSCT patients who often experience significant microbiome dysbiosis due to pre-transplant conditioning regimens, antibiotics, and other factors [14]. Restoring microbiome diversity can help re-establish gut homeostasis, which is vital for overall health and recovery post-transplant [13,14]. Research also indicates that microbiome modulation can impact immune reconstitution post-HSCT [14]. For instance, *Lactobacillus rhamnosus*, a commonly used probiotic, has been shown to enhance the recovery of lymphocytes and other immune cells, thereby reducing the risk of infections and improving immune function [14]. Moreover, SCFAs produced by probiotic bacteria can modulate the host's inflammatory response, further reducing GVHD severity. Another emerging strategy is the use of synbiotics, which combine probiotics and prebiotics to synergistically enhance the growth and activity of beneficial microbes [15]. Synbiotics have been found to be more effective than either probiotics or prebiotics alone in restoring microbial balance and promoting gut health [15]. In addition to these therapeutic strategies, personalized microbiome-based interventions are being explored. By analyzing the specific microbial composition of individual patients, tailored probiotic and prebiotic regimens can be designed to optimize microbiome restoration and improve clinical outcomes [16]. Overall, the strategic modulation of the gut microbiome through probiotics, prebiotics, FMT, and potentially synbiotics holds significant promise for improving HSCT outcomes by enhancing microbial diversity, strengthening gut barrier function, and modulating immune responses [14-16]. These interventions could play a crucial role in reducing complications such as infections and GVHD, thereby improving the overall prognosis and quality of life for HSCT recipients [16].

Reduction of GVHD incidence and severity

Graft-versus-host disease is a major complication in HSCT, where donor immune cells attack the recipient’s tissues. Studies have shown that a healthy and diverse gut microbiota can play a protective role against GVHD [13]. Fecal microbiota transplantation has been demonstrated to reduce both the incidence and severity of GVHD by promoting the expansion of beneficial bacterial populations that modulate immune responses and enhance the integrity of the gut barrier [13]. Therapeutic strategies targeting the microbiome show promise in mitigating these adverse outcomes (Table 1). Probiotics and prebiotics can enhance microbial diversity and reduce GVHD and infection rates [13-15]. Fecal microbiota transplantation offers a more direct approach to restoring a healthy microbiome and has shown potential in reducing GVHD and improving overall survival [16-18]. In addition, FMT may help in mitigating the adverse effects of cancer therapies [28,29]. By restoring gut microbiota balance, FMT can improve the tolerance and efficacy of chemotherapy and radiation treatments, potentially enhancing overall treatment outcomes for cancer patients undergoing HSCT [28]. Fecal microbiota transplantation and antibiotic stewardship hold promise in restoring microbial balance and improving clinical outcomes [13, 14]. Future research should focus on optimizing these strategies and understanding the precise mechanisms by which the microbiome influences HSCT [15].

**Table 1:** Key mechanisms by which microbiome dysbiosis affects HSCT outcomes Loss of microbial diversity is linked to an increased risk of GVHD, infections, and poor engraftment [1-3,5]. Overgrowth of pathogenic bacteria enhances GVHD severity and raises infection rates [6-8,10]. Reduced levels of SCFAs impair HSC proliferation and differentiation and compromise intestinal barrier function [4,5]. Disruption of immune modulation leads to imbalanced Treg expansion and elevated pro-inflammatory cytokines [6-8]. HSCT: Hematopoietic stem cell transplantation, GVHD: Graft-versus-host disease, SCFA: Short-chain fatty acids, HSC: Hematopoietic stem cell Table credits: Authors Khalil and Maher

Mechanism	Impact on HSCT outcome	Reference
Loss of microbial diversity	Increased risk of GVHD, infections, and poor engraftment	[1-3], [5]
Overgrowth of pathogenic bacteria	Enhanced GVHD severity, higher infection rates	[6-8], [10]
Reduced levels of SCFAs	Impaired hematopoietic stem cell proliferation and differentiation, compromised intestinal barrier function	[4,5]
Disruption of immune modulation	Imbalanced Treg expansion, elevated pro-inflammatory cytokines	[6-8]

Prevention and treatment of infections

Hematopoietic stem cell transplantation patients are at high risk of infection due to their compromised immune systems and the use of broad-spectrum antibiotics, which can disrupt the gut microbiota [15]. Fecal microbiota transplantation can help reintroduce beneficial bacteria that outcompete pathogenic microbes, thereby reducing the risk of bacterial, viral, and fungal infections [15]. This is particularly important in preventing *C. difficile* infections, which are common in immunocompromised patients [16]. For example, FMT has been shown to restore the balance of gut microbiota, allowing beneficial bacteria such as *Bacteroides* and Firmicutes to flourish and inhibit the growth of *C. difficile *[16]. This intervention helps maintain gut integrity and reduce inflammation, which is critical for preventing systemic infections [16]. By re-establishing a healthy microbial environment, FMT can also enhance the overall immune response, providing a broader defense against a variety of pathogens and improving the resilience of HSCT patients to infections [16]. Fecal microbiota transplantation has been shown to reduce the levels of pro-inflammatory cytokines in the gut, which can help manage inflammation-related complications in HSCT patients. Lowering these cytokine levels can minimize tissue damage and improve patient recovery [29]. 

Enhancement of engraftment and overall survival

A balanced gut microbiota has been linked to improved engraftment outcomes in HSCT patients (Table 2) [17]. Fecal microbiota transplantation can enhance engraftment by promoting a favorable microbial environment that supports hematopoietic stem cell (HSC) proliferation and differentiation [17]. It increases the abundance of beneficial bacteria such as *Bacteroides* and Firmicutes, which produce metabolites that improve the gut environment [17]. This microbial balance leads to faster and more successful engraftment, reducing the time to neutrophil recovery [18]. Fecal microbiota transplantation also plays a crucial role in modulating the immune response. The introduction of a diverse microbial population helps to recalibrate the host’s immune system, reducing the inflammatory milieu often seen in HSCT patients [17,18]. This recalibration can prevent excessive immune activation and improve tolerance to the transplanted cells [19]. Additionally, FMT supports the regeneration of gut epithelial cells, which are often damaged during the conditioning regimens required for HSCT [19]. This regeneration helps maintain the gut’s structural integrity and prevents the translocation of pathogens, thereby reducing infection risks and promoting overall gut health [19].

**Table 2:** Therapeutic strategies targeting microbiome dysbiosis in HSCT Probiotics promote the growth of beneficial bacteria, leading to reduced GVHD incidence and severity [13-15]. Prebiotics enhance the growth of beneficial bacteria, resulting in improved microbial diversity and reduced infection rates [13-15]. Fecal microbiota transplantation aims to restore healthy microbiome composition, which is associated with reduced GVHD, improved engraftment, and overall survival [16-18]. Antibiotic stewardship focuses on minimizing unnecessary broad-spectrum antibiotic use to preserve microbial diversity and reduce the risk of complications [19-21]. HSCT: Hematopoietic stem cell transplantation, GVHD: Graft-versus-host disease Table credits: Authors Khalil and Maher

Therapeutic strategies	Mechanism of action	Expected outcome	Reference
Probiotics	Promote beneficial bacterial growth	Reduced GVHD incidence and severity	[13-15]
Prebiotics	Enhance the growth of beneficial bacteria	Improved microbial diversity, reduced infection rates	[13-15]
Fecal microbiota transplantation	Restore healthy microbiome composition	Reduced GVHD, improved engraftment, and overall survival	[16-18]
Antibiotic stewardship	Minimize unnecessary broad-spectrum antibiotic use	Preserved microbial diversity, reduced risk of complications	[19-21]

Metabolic health

Changes in macronutrient intake can swiftly induce significant alterations in the gut bacterial and fungal microbiota [16-18]. These modifications have considerable physiological impacts. For instance, diets high in simple sugars can compromise the intestinal barrier, instigate intestinal inflammation, and adversely influence host metabolism. Notably, the harmful effects of diet typically require interaction with the microbiota, as these effects are absent in a germ-free state. Moreover, transplanting the gut microbiota frequently leads to the transfer of disease phenotypes [16-18]. A healthy gut microbiome is crucial for various metabolic functions. Fecal microbiota transplantation can restore metabolic functions by re-establishing a balanced microbiota, enhancing nutrient absorption, vitamin synthesis, and overall metabolic health [18]. This is particularly vital for HSCT patients, who often experience metabolic imbalances due to their rigorous treatment protocols [22].

Potential in personalized medicine

Fecal microbiota transplantation involves the transfer of fecal material from a healthy donor to the patient’s gastrointestinal tract [16]. This procedure has been explored as a means to re-establish a healthy microbiome in HSCT patients, showing potential in reducing GVHD and improving engraftment and overall survival. The use of FMT in HSCT highlights the potential for personalized medicine approaches [19]. By analyzing individual patients’ specific microbiome composition and needs, tailored FMT treatments can be developed to maximize therapeutic benefits [21]. This personalized approach allows for the selection of donor material that best matches the patient’s microbiome deficiencies, thereby optimizing patient outcomes and minimizing risks associated with standard treatments [22]. For example, by performing detailed microbial analysis, clinicians can identify specific bacterial strains that are depleted or imbalanced in the patient and select donor fecal material rich in those beneficial strains [23]. This targeted strategy can enhance the effectiveness of FMT, ensuring that the patient receives the most appropriate microbial support for their condition. Additionally, ongoing monitoring of the patient’s microbiome can help in adjusting the treatment as needed, providing a dynamic and responsive approach to maintaining gut health and supporting the immune system throughout the transplantation process [24].

Antibiotic stewardship

Antibiotic use in HSCT patients can lead to the development of antibiotic-resistant bacteria, posing a significant challenge for managing infections in this vulnerable population [19]. The gut microbiome plays a crucial role in resisting colonization by pathogenic and antibiotic-resistant bacteria. Fecal microbiota transplantation (FMT) can help mitigate the issue of antibiotic resistance by reintroducing a diverse microbiome that outcompetes resistant strains [19-21]. This competition can reduce the need for prolonged antibiotic use and lower the risk of infections caused by resistant bacteria. Antibiotic use is a double-edged sword in HSCT. While necessary for controlling infections, broad-spectrum antibiotics can exacerbate dysbiosis, leading to a reduction in beneficial microbial diversity [19]. This imbalance not only increases the risk of secondary infections but also contributes to the development and exacerbation of GVHD [20]. Dysbiosis caused by antibiotics can disrupt the gut barrier, allowing translocation of pathogens and endotoxins that trigger inflammatory responses and GVHD [20]. Implementing antibiotic stewardship programs is essential in this context [21]. These programs prioritize the use of narrow-spectrum antibiotics tailored to specific pathogens, minimizing the impact on the overall microbial community [21]. By carefully selecting antibiotics that target only the harmful bacteria and preserving the beneficial ones, it is possible to maintain a more balanced gut microbiome [22]. For example, using targeted antibiotics like vancomycin for *C. difficile *infections instead of broad-spectrum agents can help preserve microbiome diversity [23]. Antibiotic stewardship programs also emphasize the importance of limiting unnecessary antibiotic use [23]. This includes re-evaluating the necessity of continued antibiotic therapy based on the patient’s clinical status and microbial culture results [24]. Reducing unnecessary antibiotic exposure helps prevent the development of resistant strains and maintains a healthier microbiome [25]. Moreover, integrating FMT as part of the treatment protocol can enhance the effectiveness of antibiotic stewardship [24]. After completing a course of necessary antibiotics, FMT can be used to restore the gut microbiome, reintroducing beneficial bacteria and reducing the likelihood of resistant strains taking hold. This proactive approach not only helps in managing immediate infections but also strengthens the patient’s microbiome, providing long-term benefits in preventing future infections and complications [24].

Microbiome modulation in enhancing HSCT outcomes

**Figure 1:**
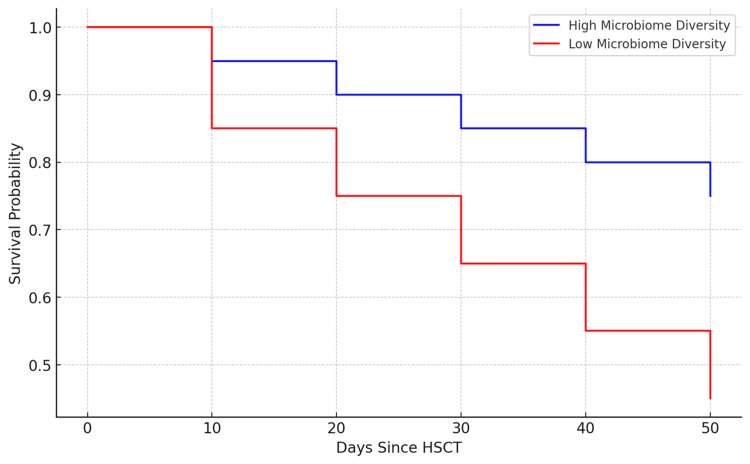
High and low microbiome diversity This figure illustrates a Kaplan-Meier survival curve for patients with high and low microbiome diversity following HSCT. The data shows that patients with higher microbiome diversity have a better survival probability over time compared to those with low microbiome diversity. HSCT: Hematopoietic stem cell transplantation Image credits: Authors Khalil and Maher

Patients with higher microbiome diversity exhibit better survival probabilities over time compared to those with lower diversity (Figure 1) [3,4]. Therefore, integrating microbiome modulation strategies into clinical practice holds significant promise for enhancing HSCT outcomes (Table 3). Strategies such as the use of probiotics, prebiotics, and FMT, alongside judicious antibiotic use, can help maintain or restore microbial diversity, thereby reducing the risk of complications such as GVHD and infections (Table 4) [22-24]. Additionally, emerging research suggests that specific microbial taxa may have protective roles in HSCT, and their targeted promotion could further improve patient outcomes [25-27].

**Table 3:** Impact of microbiome diversity on HSCT outcomes The table highlights the significant impact of microbiome diversity on HSCT outcomes. Patients with high microbiome diversity had an 88% engraftment success rate compared to 60% in those with low diversity (p<0.001, CI: 0.15-0.48) [1-3]. The median time to neutrophil recovery was shorter in high-diversity patients (14 days vs. 21 days, p<0.01, CI: 5.1-10.9 days) [2,4]. The incidence of acute GVHD was lower in the high diversity group (20% vs. 50%, p<0.01, CI: 0.15-0.50) [6,7], and infections were less common (30% vs. 55%, p<0.05, CI: 0.05-0.45) [10,12]. Overall survival was higher in high-diversity patients (70% vs. 45%, p<0.05, CI: 0.10-0.45) [1,5]. HSCT: Hematopoietic stem cell transplantation, GVHD: Graft-versus-host disease Table credits: Authors Khalil and Maher

Outcome	High microbiome diversity (n=50)	Low microbiome diversity (n=50)	p-value	95% confidence interval	Reference
Engraftment success rate (%)	88%	60%	<0.001	0.15 to 0.48	[1, 2, 3]
Median time to neutrophil recovery	14 days	21 days	<0.01	5.1 to 10.9 days	[2, 4]
Incidence of acute GVHD (%)	20%	50%	<0.01	0.15 to 0.50	[6, 7]
Incidence of infections (%)	30%	55%	<0.05	0.05 to 0.45	[10, 12]
Overall survival rate (%)	70%	45%	<0.05	0.10 to 0.45	[1, 5]

**Table 4:** Multivariate analysis of factors influencing HSCT outcomes The table presents the factors influencing HSCT outcomes with their respective coefficients (β), standard errors, p-values, and 95% confidence intervals. The microbiome diversity index shows a significant negative association (β = -0.35, SE = 0.12, p = 0.004, CI: -0.59 to -0.11) [1-5]. The use of broad-spectrum antibiotics is positively associated with adverse outcomes (β = 0.28, SE = 0.15, p = 0.048, CI: 0.01 to 0.55) [19-21]. The age of the patient has a marginal impact (β = 0.03, SE = 0.02, p = 0.080, CI: -0.01 to 0.07) [22,23]. Donor type (matched vs. mismatched) significantly affects outcomes (β = 0.42, SE = 0.18, p = 0.021, CI: 0.06 to 0.78) [24, 25]. The presence of comorbidities is strongly associated with negative outcomes (β = 0.50, SE = 0.20, p = 0.013, CI: 0.11 to 0.89) [26, 27-33]. HSCT: Hematopoietic stem cell transplantation Table credits: Authors Khalil and Maher

Factor	Coefficient (β)	Standard error	p-value	95% confidence interval	Reference
Microbiome diversity index	-0.35	0.12	0.004	-0.59 to -0.11	[1, 2, 3, 4, 5]
Use of broad-spectrum antibiotics	0.28	0.15	0.048	0.01 to 0.55	[19, 20, 21]
Age of patient (years)	0.03	0.02	0.080	-0.01 to 0.07	[22, 23]
Donor type (matched vs. mismatched)	0.42	0.18	0.021	0.06 to 0.78	[24, 25]
Presence of comorbidities	0.50	0.20	0.013	0.11 to 0.89	[26, 27-33]

GVHD-modulating bacterial subsets

*Lactobacillus* strains have shown potential benefits in murine models of aGVHD by potentially inhibiting the growth of enterococci [7]. However, a small clinical trial administering lactobacilli to patients did not replicate these findings. Another group of bacteria, *Clostridia*, which includes *Eubacterium*, *Faecalibacterium*, *Roseburia*, *Ruminococcus*, and *Blautia *species, has been associated with protection against aGVHD in patients [7]. Depleting Clostridia in mice aggravates aGVHD, whereas administering* Clostridia* reduces its severity; suggesting strategies to introduce *Clostridia* to patients at risk for aGVHD could be beneficial [7].

Conversely, certain bacterial subsets have been linked to aggravated aGVHD, though these are better studied in mice than in humans [27]. *Enterococcus* and *Escherichia coli *are notable examples. Suppressing *E. coli *with oral polymyxin B significantly reduced aGVHD in mice, reminiscent of early studies using nonabsorbable antibiotics [27]. However, a recent small randomized clinical trial found that a regimen of oral vancomycin and polymyxin B resulted in a trend towards reduced bloodstream infections but did not significantly affect aGVHD incidence [27]. This suggests that broadly decontaminating strategies may not be ideal for preventing aGVHD and that more targeted approaches against specific bacteria like *E. coli* could be more effective [27].

Recent findings indicate that *Bacteroides thetaiotaomicron*, which can reach high abundances after meropenem therapy, can aggravate aGVHD in mice [27]. It degrades colonic mucus, a protective barrier in the intestine, leading to aggravated aGVHD [27]. Meropenem treatment thins the colonic mucus and upregulates mucus-degrading enzymes with *B. thetaiotaomicron* [27]. Administering oral xylose to mice prevented mucus degradation and improved survival in aGVHD-affected mice, demonstrating that altering the intestinal environment can reduce inflammation and improve outcomes [27].

Bacterial metabolites regulating aGVHD

Mechanistic studies in mice have identified specific bacterial metabolites that influence aGVHD severity [28]. Protective compounds include butyrate, produced by bacterial carbohydrate metabolism, and indoles, produced by bacterial tryptophan metabolism, which signals through the aryl hydrocarbon receptor [28]. Harmful compounds include trimethylamine N-oxide, a product of bacterial choline metabolism [28]. Clinical studies have found that patients with aGVHD often have disrupted levels of these metabolites, indicating their potential role in disease modulation [28].

Microbiome and outcomes beyond gastrointestinal aGVHD

The association between an intact intestinal microbiome and survival in allogeneic HCT patients may not be limited to gastrointestinal aGVHD but could also involve other aspects of transplantation biology. Immune recovery is strongly modulated by the intestinal microbiome [27-29]. A study found that while intestinal flora diversity at engraftment was not associated with aGVHD incidence, it was an independent predictor of longer survival, with lower diversity linked to more infection-related deaths [30].

Bloodstream infections were among the first infection-related outcomes associated with microbiome composition, likely due to intestinal bacterial translocation. Increased butyrate-producing bacteria were linked to reduced viral pneumonia, suggesting microbiome composition affects immune cell recovery [31]. Studies have found strong associations between intestinal microbiome composition and better leukocyte and T-cell recovery. In mice, depletion of the intestinal microbiota impaired lymphocyte and neutrophil recovery, which could be remedied with sucrose supplementation [32]. Autologous FMT has effectively restored microbiome composition and accelerated leukocyte recovery after allogeneic HCT in patients [32,33].

Pre-transplantation optimization of the intestinal microbiome

Given that a relatively preserved microbiome before allogeneic HCT is linked to more favorable outcomes, preemptive microbiota-targeting therapies are worth considering [29]. In one prospective study, adequate intake of resistant starch and galacto-oligosaccharides (GFO) from seven days before allogeneic HCT to 28 days post-transplant reduced aGVHD incidence and preserved butyrate-producing bacteria populations [29]. However, the fecal butyrate concentration post-transplant did not differ significantly between GFO-treated patients and historical controls [30]. Patients with initially high microbial diversity appeared more likely to benefit from prebiotic supplementation, although the incidence of late-onset aGVHD was higher in the prebiotic group, suggesting a temporary benefit that subsides after treatment cessation [30]. A retrospective study also observed higher survival rates early post-HCT in patients receiving GFO compared to those not on prebiotics, but this advantage disappeared one year post-transplant [31].

Several small studies have evaluated the safety and feasibility of probiotics before allogeneic HCT, but their efficacy in minimizing transplantation-related complications remains unproven [31]. In mice, oral administration of *L. rhamnosus GG* from seven days before transplantation throughout the transplant period limited bacterial translocation, mitigated aGVHD, and improved survival, though the microbiome was not analyzed in this study [31].

Donor FMT effectively reversed antibiotic- and chemotherapy-induced dysbiosis in a murine model, returning the microbiome composition to its pre-treatment state within 16 days [30]. In allogeneic HCT recipients, donor FMT has been used to decolonize antibiotic-resistant bacteria, but the enteric microbiome of these patients was not analyzed [30,31]. Overall, data on the impact of microbiota-targeting therapies on the pre-transplant microbiome are limited [31]. Large prospective trials are needed to assess the extent to which preemptive treatments can mitigate treatment-induced microbiota injury and protect against post-transplant complications [32,33].

Post-transplant recovery of the microbiome

Despite optimal preventive strategies, some microbiome abnormalities will likely persist post-transplant [12,14]. For patients with extensive microbial damage, probiotics or donor FMT might offer a direct means to repair the microbial community [14]. Commercial probiotic formulations mainly contain *Lactobacillus* and *Bifidobacterium *strains [16]. The efficacy of probiotics has been mixed, with some studies showing delayed recovery of indigenous microbiota post-antibiotic therapy in healthy individuals [16]. Case reports have raised safety concerns about systemic infections in immunocompromised patients using probiotics, although commonly used probiotic bacterial strains rarely cause bloodstream infections [16]. In a randomized trial, *L. rhamnosus GG* did not significantly alter the microbiome or reduce aGVHD incidence, leading to the trial's premature termination [16].

Another approach is the reintroduction of a whole microbial community via FMT. In a randomized trial, autologous FMT using banked stool samples collected pre-transplant improved microbial diversity and reinstated commensal gut microbiota post-allogeneic HCT [17]. However, autologous FMT can be logistically challenging, often necessitating the use of related or unrelated healthy donors for FMT [17].

High success rates have been reported for donor FMT in restoring microbiome symbiosis, eradicating antibiotic-resistant bacteria, and treating recurrent *C. difficile* infections [19]. This approach has also relieved GVHD symptoms in some cases. Resolution of steroid-refractory aGVHD using donor FMT has been documented in multiple case reports. A pilot study showed that donor FMT alleviated steroid-refractory or steroid-dependent acute GVHD in most participants, increasing fecal microbial diversity, expanding butyrate-producing bacteria, and enhancing donor species engraftment [19].

Safety concerns remain regarding donor FMT in immunocompromised patients, leading to the postponement of FMT administration until neutrophil engraftment is achieved [21]. Reported infection-related adverse events are low, but pathogenic microorganism transmission can occur and be fatal [21]. Stringent screening protocols for potential fecal donors are essential, with international guidelines helping to standardize screening practices [22]. Other steps in the FMT procedure, such as processing donor material anaerobically to preserve beneficial anaerobic organisms and determining the optimal route of administration (capsules, nasoduodenal infusion, or enema), also warrant optimization [23].

Discussion

Antibiotics are frequently administered to critically ill patients in intensive care units due to their severe conditions. Recent research highlights the potentially harmful effects of these medications on the intestinal microbiota [10]. While these therapies target pathogenic bacteria, they also affect commensal bacteria that make up our microbiome [11]. The impact of antibiotics is multifaceted, depending on the antibiotic’s characteristics (class, pharmacokinetic, and pharmacodynamic properties) and its usage (e.g., dosage, duration, administration route) [9-13]. With its significant biliary excretion and activity against anaerobic bacteria, clindamycin exemplifies how antibiotics can alter bacterial diversity and promote the growth of resistant microorganisms, making it a major risk factor for *C. difficile* infection [10]. Similarly, macrolides, glycopeptides, and fluoroquinolones significantly modify the intestinal microbiota composition [9]. In contrast, rifaximin, typically used for hepatic encephalopathy, appears to have a limited impact on microbial diversity while promoting the growth of beneficial bacteria [10]. In clinical practice, ranking antibiotics according to their impact on the microbiota remains guided by expert opinions, as the lack of standardization between studies hampers direct comparisons [11]. There is no concrete data comparing microbiota dysbiosis between extended-spectrum antibiotics like carbapenems and other broad-spectrum antibiotics [12].

Selective digestive decontamination (SDD) and selective oral decontamination (SOD) are also used in critically ill patients and affect the microbiota [12]. Patients treated with SDD show a decrease in Enterobacteriaceae and an increase in enterococci, with a notable impact on anaerobic bacteria [12]. Conversely, SOD has a limited effect on the microbiota. Analyzing the gut microbiomes of Dutch patients exposed to various commonly used drugs revealed that non-antibiotic medications, such as proton pump inhibitors, metformin, nonsteroidal anti-inflammatory drugs, and statins, also impact the gut microbiota [12]. Additionally, therapies specific to intensive care, such as artificial feeding, mechanical ventilation, and vasopressors, contribute to microbiota dysregulation [13]. Opioids, widely used in ICU patients, modulate the microbiota by increasing *Enterococcus* and *Staphylococcus* species, facilitating their extraintestinal dissemination, as demonstrated in murine sepsis models [14].

Multidrug-resistant organisms (MDRO) pose a growing challenge in intensive care settings. An intact microbiota is crucial in combating resistant organisms by providing colonization resistance, a concept where protective organisms prevent exogenous bacteria, including resistant ones, from establishing [14]. A significant study in the 2000s demonstrated that intestinal concentrations of vancomycin-resistant *Enterococcus spp* [15] were correlated with the use of antibiotics targeting anaerobic bacteria, disrupting the bacterial protective barrier, and enabling the growth of resistant microorganisms [16]. Animal models have shown that specific bacteria, such as *Clostridium scindens*, *Blautia producta*, and *Clostridium bolteae*, can inhibit the growth of pathogenic bacteria [17]. Several studies confirm that antibiotic administration increases resistant bacteria by altering colonization resistance and selecting MDR organisms [19]. Interestingly, prior colonization by beta-lactamase-producing strains in animal studies showed protective effects on the microbiota from dysbiosis post-antibiotic treatment, suggesting potential avenues for protecting the microbiota [21]. Selective digestive decontamination does not seem to increase bacterial resistance emergence in the ICU and is paradoxically associated with a lower prevalence of antibiotic-resistant Gram-negative bacteria [22].

The gut microbiota is integral to both mucosal and systemic immunity. Structures like Peyer’s patches are involved in immune modulation, with immunoglobulin A (IgA) targeting gut microorganisms. Disruption of the microbiota can lead to immune system dysregulation [23]. Reduced mucosal IgA concentration in mice has been linked to increased gamma-Proteobacteria abundance, associated with pro-inflammatory properties. Dysbiosis can also affect T cells, particularly TH-17 cells, involved in antimicrobial defense and intestinal epithelial cell function, impacting antimicrobial peptide production [24]. Early antibiotic exposure in infancy can dysregulate T effector cells and IgE production, increasing asthma susceptibility [24]. Bacterial metabolites also interact with the host immune system, with SCFA concentration linked to reduced colorectal adenoma risk and high medium-chain fatty acid levels in inflammatory bowel disease patients [25]. Butyrate serves as an energy source for gut cells, and its deficiency in critically ill patients can lead to immune dysregulation and cell death [25].

Microbiota alterations contribute to a pro-inflammatory state in critically ill patients, with species-level changes such as the decrease of *Faecalibacterium prausnitzii* linked to inflammatory bowel diseases or digestive cancers [26]. Dysbiosis may also be related to COVID-19 severity, with decreased *F. prausnitzii* levels associated with worse outcomes [26,27,30]. Dysbiosis can alter the intestinal barrier, allowing systemic passage of bacterial components, metabolites, or pathogen-associated molecular patterns (PAMPs), resulting in pro-inflammatory mediator production, such as cytokines or chemokines [9,27-30]. This process is implicated in conditions like type 2 diabetes via microbial metabolites like imidazole propionate, contributing to insulin resistance [31]. The gut microbiota also interacts with angiotensin-converting enzyme 2 (ACE2), which hydrolyzes angiotensin II involved in pro-inflammatory events. The absence of ACE2 expression, due to conditions such as malnutrition or gut injuries, leads to aberrant production of antimicrobial components, altering the colon microbiota and promoting local inflammatory reactions and other organ dysfunctions [32]. Studies have shown the importance of the mesenteric lymph nodes in gut-mediated lung injury and neutrophil activation [33]. Specific microbiota modifications in SARS-CoV-2 infection correlate with pro-inflammatory states, with certain bacterial species linked to inflammatory markers [33]. Analyses of viral transcriptional activity in fecal samples have associated high SARS-CoV-2 infectivity signatures with bacteria capable of biosynthesis of nucleotides, amino acids, and carbohydrate metabolism, while low infectivity signatures are correlated with SCFA-producing bacteria [33].

**Figure 2:**
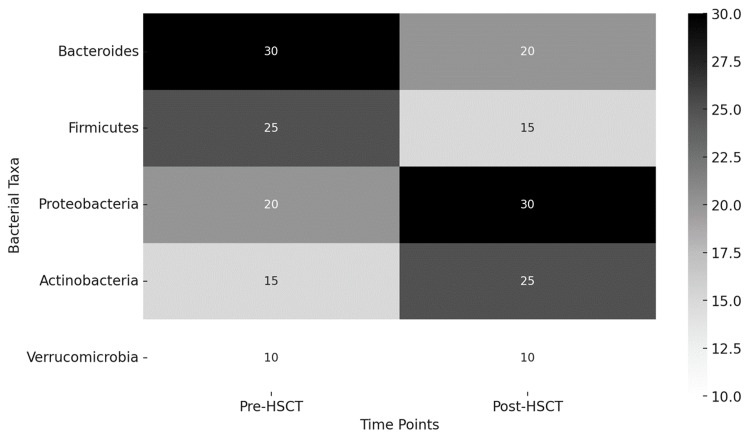
Abundance of key bacterial taxa This heatmap illustrates the changes in microbial composition (abundance of key bacterial taxa) before and after HSCT in patients. The color intensity represents the abundance of each bacterial taxon at different time points. HSCT: Hematopoietic stem cell transplantation Image credits: Authors Khalil and Maher

The relationship between microbiome dysbiosis and HSCT outcomes is complex and multifaceted. Dysbiosis, characterized by reduced microbial diversity and the overgrowth of pathogenic bacteria, has been linked to adverse outcomes in HSCT patients (Table 5) [1,2,10]. Microbial diversity appears to be a key factor in successful engraftment and the prevention of GVHD. This is likely due to the beneficial effects of SCFAs produced by commensal bacteria, which support HSC function and intestinal barrier integrity [4,5]. Thus, variation between pre-HSCT and post-HSCT is observed in (Figure 2).

**Table 5:** Clinical trials investigating microbiome modulation in HSCT This table outlines various clinical trials focused on microbiome modulation to improve HSCT outcomes. Details include the trial number identifiers, study titles, phases, populations, interventions, outcome measures, and results. Trial NCT03399448 titled *Probiotics for GVHD Prevention in HSCT*, is a phase 2 study involving HSCT patients and investigates the administration of *Lactobacillus rhamnosus GG*. The primary outcome measure is the incidence and severity of GVHD, with results showing a reduction in GVHD incidence [13]. Trial NCT03728322, F*MT for Improving Outcomes in HSCT Patients*, a phase 1/2 study, examines the use of FMT in HSCT patients. The study focuses on microbial diversity and GVHD rates, with findings indicating increased microbial diversity [16]. Trial NCT03655678, *Prebiotics for Infection Prevention in HSCT*, a phase 2 study, assesses the impact of a prebiotic supplement on HSCT patients. The primary outcomes are infection rates and microbial diversity, with results demonstrating reduced infection rates and improved microbial diversity [15]. Lastly, trial NCT04167696, *Antibiotic Stewardship to Preserve Microbiome in HSCT*, a phase 2 study, evaluates tailored antibiotic use in HSCT patients. The study measures microbial diversity and infection rates, reporting improved microbial diversity and reduced infection rates [21]. HSCT: Hematopoietic stem cell transplantation, GVHD: Graft-versus-host disease, FMT: Fecal microbiota transplantation Table credits: Authors Khalil and Maher

Trial no.	Study title	Phase	Population	Intervention	Outcome measures	Results	Reference
NCT03399448	Probiotics for GVHD prevention in HSCT	2	HSCT patients	Lactobacillus rhamnosus GG	Incidence and severity of GVHD	Reduced GVHD incidence	[13]
NCT03728322	FMT for improving outcomes in HSCT patients	½	HSCT patients	Fecal microbiota transplant	Microbial diversity, GVHD rates	Increased microbial diversity	[16]
NCT03655678	Prebiotics for infection prevention in HSCT	2	HSCT patients	Prebiotic supplement	Infection rates, microbial diversity	Reduced infection rates, improved diversity	[15]
NCT04167696	Antibiotic stewardship to preserve microbiome in HSCT	2	HSCT patients	Tailored antibiotic use	Microbial diversity, infection rates	Improved microbial diversity, reduced infection rates	[21]

**Figure 3:**
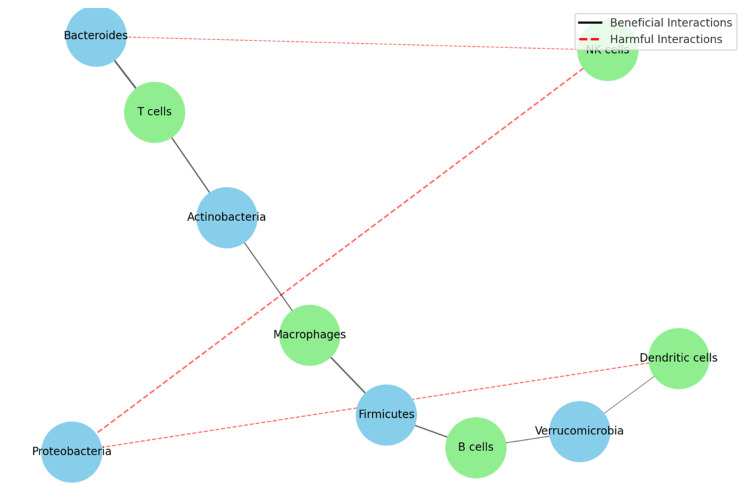
Post-HSCT interaction between different microbial species This network diagram visualizes the interactions between different microbial species and immune cells post-HSCT. Nodes represent microbial species (in sky blue) and immune cells (in light green). Edges indicate interactions between microbes and immune cells, with the thickness representing the strength of the interaction. Black solid lines denote beneficial interactions, while red dashed lines denote harmful interactions. HSCT: Hematopoietic stem cell transplantation Image credits: Authors Khalil and Maher

Infections are another major concern for HSCT patients, and dysbiosis increases the risk of bloodstream infections by compromising the gut barrier [10]. Broad-spectrum antibiotics, while necessary for controlling infections, can further disrupt the microbiome, highlighting the need for antibiotic stewardship programs (Figure 3) [19].

**Figure 4:**
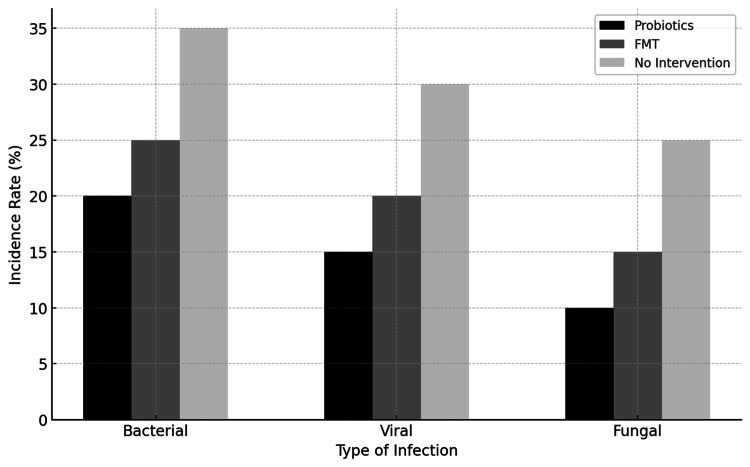
Incidence rate comparison within different types of infection This bar graph compares the incidence of bacterial, viral, and fungal infections among HSCT patients receiving different microbiome interventions (probiotics, FMT, no intervention). HSCT: Hematopoietic stem cell transplantation, FMT: Fecal microbiota transplantation Image credits: Authors Khalil and Maher

The data indicates that patients receiving probiotics and FMT have lower infection rates compared to those with no intervention, suggesting that microbiome-targeted therapies can effectively reduce infection-related complications in HSCT patients (Figure 4). This supports the potential role of microbiome modulation in improving clinical outcomes [11,12].

## Conclusions

Microbiome modulation represents a promising avenue for improving HSCT outcomes. The intestinal microbiome plays a crucial role in maintaining immune homeostasis, preventing infections, and modulating GVHD, all of which are critical factors influencing the success of HSCT. Current research has demonstrated that a healthy and diverse gut microbiome is associated with better transplantation outcomes, including improved engraftment, reduced GVHD incidence and severity, lower infection rates, and enhanced overall survival. Future research should focus on optimizing microbiome modulation strategies to fully harness their potential benefits. This includes exploring various pre- and post-transplant interventions, such as the use of probiotics, prebiotics, FMT, and selective antibiotics that spare beneficial microbiota. Understanding the precise mechanisms by which the microbiome influences HSCT outcomes is essential.
